# Antimetastatic Properties of Prodigiosin and the BH3-Mimetic Obatoclax (GX15-070) in Melanoma

**DOI:** 10.3390/pharmaceutics15010097

**Published:** 2022-12-28

**Authors:** Margarita Espona-Fiedler, Pilar Manuel-Manresa, Cristina Benítez-García, Pere Fontova, Roberto Quesada, Vanessa Soto-Cerrato, Ricardo Pérez-Tomás

**Affiliations:** 1 Department of Pathology and Experimental Therapeutics, Faculty of Medicine and Health Sciences, Universitat de Barcelona, 08907 L’Hospitalet de Llobregat, Spain; 2 Molecular Signalling, Oncobell Program, Institut d’Investigació Biomèdica de Bellvitge (IDIBELL), 08908 L’Hospitalet de Llobregat, Spain; 3 Department of Chemistry, Universidad de Burgos, 09001 Burgos, Spain

**Keywords:** metastasis, obatoclax, prodigiosin, prodiginines, BH3-mimetic, melanoma, migration

## Abstract

Metastasis is the primary cause of death in cancer patients. Many current chemotherapeutic agents only show cytotoxic, but not antimetastatic properties. This leads to a reduction in tumor size, but allows cancer cells to disseminate, which ultimately causes patient death. Therefore, novel anticancer compounds with both effects need to be developed. In this work, we analyze the antimetastatic properties of prodigiosin and obatoclax (GX15-070), anticancer drugs of the Prodiginines (PGs) family. We studied PGs’ effects on cellular adhesion and morphology in the human primary and metastatic melanoma cell lines, SK-MEL-28 and SK-MEL-5, and in the murine melanoma cell line, B16F10A. Cell adhesion sharply decreased in the treated cells, and this was accompanied by a reduction in filopodia protrusions and a significant decrease in the number of focal-adhesion structures. Moreover, cell migration was assessed through the wound-healing assay and cell motility was severely inhibited after 24 h of treatment. To elucidate the molecular mechanisms involved, changes in metastasis-related genes were analyzed through a gene-expression array. Key genes related to cellular invasion, migration and chemoresistance were significantly down-regulated. Finally, an in vivo model of melanoma-induced lung metastasis was established and significant differences in lung tumors were observed in the obatoclax-treated mice. Altogether, these results describe, in depth, PGs’ cellular antimetastatic effects and identify in vivo antimetastatic properties of Obatoclax.

## 1. Introduction

Metastasis is a complex process, which occurs through several sequential steps and that involves local invasion, intravasation, transport through the vasculature, arrest at a distant site, extravasation and microenvironment adaptation to grow in a secondary site [1]. Although most well-confined primary tumors can be cured by surgical resection and adjuvant therapy, metastatic disease is largely incurable, being responsible for most of human cancer-related deaths [2]. This is mainly due to the systemic nature of the disease and the development of resistance to conventional chemotherapeutic agents by disseminated tumor cells. This is particularly the case for melanoma, an extremely aggressive disease with high metastatic potential and an elevated resistance to cytotoxic agents [3,4]. Metastatic melanoma, although being much less common than other cutaneous malignancies, has a very bad prognosis, with a five-year survival rate of 27.3% [5]. Before 2010, the DNA methylating compound dacarbazine was considered the reference drug, although complete responses occurred in less than 5% of cases and were short in duration [6]. Since 2011, several novel agents have been approved for this indication, such as the immunomodulating agents tebentafusp, ipilimumab or anti-PD1 antibodies, as well as several targeted drugs, including selective BRAF and MEK inhibitors, among others [7,8,9]. These targeted therapies have improved progression-free and overall survival rates, but only in a selected subset of patients.

Despite the clinical relevance of metastatic disease, the lack of effective drugs for the majority of patients limits its treatment; therefore, it is crucial to focus our efforts on the identification and development of novel therapeutic agents which possess not only antitumor, but also antimetastatic properties. In this view, small molecules called prodiginines (PGs) are secondary metabolites of bacterial origin characterized by a shared pyrrolylpyrromethene core [10]. These compounds possess different pharmacological properties and have emerged as novel anticancer agents for a wide panel of cancer cells from different origins [11,12,13]. Prodigiosin (PG), a member of this chemical family, has also been shown to possess antimetastatic properties in vivo [14]. Inspired by this structure, the synthetic prodiginine analog, obatoclax (OBX, GX015-070), was developed and has been evaluated in clinical trials for the treatment of different cancer malignancies, such as leukemia, lymphomas or small-cell lung cancer [15]. Obatoclax has shown in vitro effects on the cell migration of hepatocarcinoma cells, as well as the prevention of tumor onset in a hepatoblastoma mouse model [16,17], but no potential in vivo antimetastatic effects on melanoma have been evaluated yet. The potent cytotoxic effects of both molecules are mediated by several mechanisms of action. These molecules have been identified as effective inhibitors of the mammalian target of rapamycin (mTOR) complexes [18], which are central regulators of cellular metabolism in malignant cells. Inhibitors that target this pathway have shown encouraging results in the treatment of certain patients with solid tumors [19]. Moreover, PGs have also been described as BH3-mimetic molecules [20,21], which are small molecules that bind to the hydrophobic groove in anti-apoptotic BCL-2 family members and trigger the release of pro-apoptotic proteins, such as BAX or BAK, sensitizing cancer cells to apoptosis. Interestingly, this class of drugs has displayed additive effects in combination with other chemotherapeutics [22,23,24,25].

Due to the promising antitumor properties of PGs, especially OBX, which has been assayed in clinical trials, we aim to evaluate in the present work the potential antimetastatic properties of PGs in vitro and in vivo in a melanoma mouse model and try to elucidate the cellular and molecular mechanisms that trigger this ability.

## 2. Materials and Methods

### 2.1. Reagents

**Figure 1:**
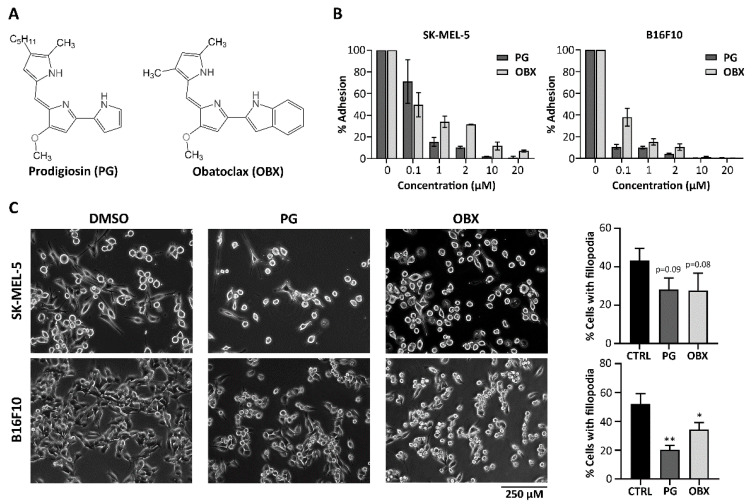
Adhesion and morphology of cancer cells after treatment with prodigiosin (PG) and obatoclax (OBX). (**A**) Chemical structures of PG and OBX. (**B**) SK-MEL-5 or B16F10 cells treated with different concentrations of PG or OBX for 16 h were detached and reseeded and cellular-adhesion capacity was quantified. (**C**) SK-MEL-5 or B16F10 cells filopodia were evaluated and quantified after 24 h of treatment with PG or OBX. Representative images from three independent experiments are shown. Figure shows mean ± SD. Statistical differences against control (CTRL) are shown as ** *p* < 0.01 and * *p* < 0.05.

Prodigiosin (PG, 4’-methoxy-5’-((5-methyl-4-pentyl-1H-pyrrol-2-yl)methylene)-1H,5’H-2,2’-bipyrrole) and obatoclax (2-(2-((3,5-dimethyl-1H-pyrrol-2-yl)methylene)-3-methoxy-2H-pyrrol-5-yl)-1H-indole) were synthesized by Dr. Roberto Quesada from the University of Burgos according to published procedures (Figure 1A) [26]. The compounds were characterized spectroscopically and the purity was estimated to be ≥95% by H qNMR. All stock solutions were diluted in DMSO and stored at −20 °C.

### 2.2. Cell Lines and Culture Conditions

Human melanoma SK-MEL-5 and SK-MEL-28 and mouse melanoma B16F10 cell lines were purchased from American Type Culture Collection (Manassas, VA, USA). The SK-MEL-5 cells were established from a metastatic site (axillary lymph node) of a patient with malignant melanoma and human melanoma SK-MEL-28 cells were established from the skin tissue of a malignant-melanoma patient. Human oral squamous carcinoma HN4 cell line was kindly provided by Dr. Silvio Gutkind (NIDCR), Bethesda, MD, USA. SK-MEL-5, SK-MEL-28 and HN4 cells were cultured in Dulbecco’s Modified Eagle’s Medium (DMEM), whereas B16F10 cells were cultured in RPMI medium (Biological Industries, Beit Haemek, Israel). All cell lines were supplemented with 10% heat-inactivated fetal bovine serum (FBS; Life Technologies, Carlsbad, CA, USA), 100 U/mL penicillin, 100 µg/mL streptomycin, and 2 mM L-glutamine, all from Biological Industries. Cells were grown at 37 °C in a 5% CO_2_ atmosphere.

### 2.3. Cell-Adhesion Assay

The SK-MEL-5 cells (2 × 10^5^ cells/mL) or B16F10 cells (1 × 10^5^ cells/mL) were seeded and after 24 h they were treated or not with a range of concentrations from 0.1 to 20 µM of PG or OBX for 16 h. Next, they were resuspended and seeded into a 96-well cell-culture plate. Subsequently, they were incubated for 2 h (SK-MEL-5 cells) or 5 h (B16F10 cells) at 37 °C and washed three times with PBS to remove floating cells and keep only the cells that were able to adhere to the plate. Finally, 10 µM of 3-(4,5-dimethylthiazol-2-yl)-2,5-diphenyltetrazolium bromide (MTT, Sigma-Aldrich, St. Louis, MO, USA) was added to each well and they were incubated for an additional 4 h. Media was aspirated and the formazan precipitate was dissolved with 100 µL of DMSO. The absorbance at 570 nm was measured on a Multiskan FC multiwell plate reader (Thermo Fisher Scientific Inc., Waltham, MA, USA). Experiments were performed twice in triplicates and cell adhesion was shown as a percentage of adherent non-treated cells.

### 2.4. Time-Lapse Microscopy

The SK-MEL-5 (2 × 10^5^ cells/mL) or B16F10 cells (1 × 10^5^ cells/mL) were seeded and after 24 h they were treated with DMSO, PG or OBX at 100 nM (SK-MEL-5) or 1 µM (B16F10) for 24 h. Monitored cells and filopodia structures were quantified at the end point in all conditions. Images were obtained with ZEISS AXIO-Observer microscope (Carl Zeiss Microscopy GmbH, Jena, Germany) under controlled conditions of temperature and CO_2_.

### 2.5. Immunofluorescence

The SK-MEL-28 and SK-MEL-5 cells (2 × 10^5^ cells/mL) were grown on coverslips for 24 h. Next, they were treated with DMSO, PG or OBX for 3 h and fixed with 4% paraformaldehyde (PFA) for 10 min. Permeabilization with PBS-Triton X-100 (0.5%) was performed during 5 min, after which cells were blocked with 1% (*w*/*v*) BSA in PBS for 15 min. Cells were incubated with primary antibody anti-vinculin (dilution 1:400, Sigma-Aldrich) for 30 min at 37 °C. Cells were then incubated with secondary antibody Cy3 goat anti-mouse IgG (Jackson ImmunoResearch, West Grove, PA, USA) diluted 1:100 and mixed with BODIPY FL Phallacidin (Invitrogen, Carlsbad, CA, USA) diluted 1:50. After incubation for 30 min at 37 °C, Mowiol mounting media (Sigma-Aldrich) was added. Preparations were dried in the dark at 4 °C. Focal-adhesion images were obtained with epifluorescence Nikon E800 microscope (Nikon Europe BV, Badhoevedorp, The Netherlands) and quantified. Results were performed in triplicate and representative images are shown. Number of focal adhesions was quantified and results (mean ± S.D.) are shown as a percentage of non-treated cells.

### 2.6. Wound-Healing Assay

The B16F10 or HN4 cells were grown in complete media to 100% confluence in 12-well dishes and a scratch was performed with a sterile tip. Next, cells were washed with PBS and DMEM (HN4 cells) or RPMI (B16F10 cells) with 10% or 1% FBS was added. Mitomycin C (2 µg/mL, Sigma-Aldrich) was added under certain conditions for 2 h after serum deprivation. Cells were treated with DMSO (vehicle) or PGs at 100 nM (HN4 cells) or 200 nM (B16F10 cells) for 24 h. Images were obtained with Leica DM IRB microscope (Leica Microsystems, Wetzlar, Germany) attached to an Olympus DP70 camera (Olympus Corporation, Tokyo, Japan). Results are shown as a percentage of the invaded area resulting from three independent experiments. Areas were analyzed and quantified with Image J v1.52 (National Institutes of Health, Bethesda, MD, USA) by a wound-healing-assay plugin reported previously [27].

### 2.7. Gene-Expression Profiling

The SK-MEL-5 cells (2 × 10^5^ cells/mL) were treated with DMSO or PG at 0.5 µM for 16 h and RNA extraction was performed with RNeasy® mini kit (Qiagen, Valencia, CA, USA). In order to obtain the cDNA, 1 µg RNA was used for reverse transcription with the High-Capacity cDNA Reverse Transcription kit (Applied Biosystems, Foster, CA, USA). TaqMan® Low-Density Array (TLDA) micro fluidic cards (Applied Biosystems), designed for assessing the expression of 87 known human tumor-metastasis-related genes and 8 endogenous controls, were used. The experiment was then performed according to manufacturer’s instructions using the 7900HT Fast Real-Time PCR system (Applied Biosystems). Each sample was run in duplicate. The DataAssist^TM^ software (Applied Biosystems) was used to determine the most appropriate endogenous control gene for our experimental conditions and to analyze the results. Beta-2-microglobulin was found to be the most stable gene after PG treatment.

### 2.8. Western-Blot Analysis

The SK-MEL-5 cells (2 × 10^5^ cells/mL) were treated with PG or OBX at 500 nM for different time periods. Adherent and floating cells were collected, washed twice and lysed for 15 min at 4 °C in RIPA buffer (0.1% SDS, 1% NP-40, 0.5% sodium deoxycholate, 50 mM NaF, 40 mM β-glycerophosphate, 200 µM sodium orthovanadate, 1 mM phenylmethylsulfonyl fluoride and complete mini-protease-inhibitor cocktail (Roche, Basel, Switzerland)). Protein concentration was determined with the BCA protein assay (Pierce, Rockford, IL, USA) and 50 µg of protein extracts were separated by SDS-PAGE and transferred to Immobilon-P membranes (Millipore, Bedford, MA, USA). Immunoblots were incubated with anti-twist1 (#AV37997, Sigma-Aldrich), anti-vimentin (#3932, Cell Signaling Technology, Danvers, MA, USA), anti-MMP1 (#IM35, Calbiochem, Merck KGaA, Billerica, MA, USA) or anti-actin (sc-1616, Santa Cruz Biotechnology, Dallas, TX, USA) antibodies, according to manufacturer’s instructions. Antibody binding was detected with secondary antibodies conjugated to horseradish peroxidase (Santa Cruz Biotechnology) and the ECL detection kit (Amersham, Buckinghamshire, UK). Actin was used as a gel-loading control. Representative results of independent experiments are shown.

### 2.9. Effects on Metastasis in Vivo

All animal procedures were approved by the Ethics Committee for Animal Experimentation of the University of Barcelona and the Autonomic Ethic Committee in accordance with Spanish and EU regulations (reference 6684). Six-week-old female Balb/c mice were purchased from Janvier Labs (St Berthevin Cedex, France) and were housed and maintained in good conditions at the animal facilities. The 1 × 10^5^ B16F10 cells in 1X PBS were inoculated by tail-vein injection and next day, mice were divided into 3 groups and intraperitoneally treated with vehicle (physiological saline solution with 0.4% (*v*/*v*) Tween-80), 2 or 4 mg/Kg OBX for 10 days. Day 16, mice were sacrificed, lungs were excised and number of visible tumor nodules per mouse was counted, while % of tumor area per lung was calculated with ImageJ software.

### 2.10. Tissue Processing and Hematoxylin-Eosin Staining

Lung samples were processed in a tissue processor, Shandun Citadel 1000 (Thermo Fisher Scientific), as follows; two washing steps in PBS pH 7.4 for 2 h and 1 h 30 min, followed by gradual dehydration in solutions with increasing concentrations of ethanol: 1 h 30 min at 30%, 2 h at 70%, 2 h at 96% and twice in absolute ethanol for 2 h each. Next, the samples were cleared twice in xylene for 1 h 30 min and 2 h and then infiltrated in paraffin wax (PanReac Applichem, Castellar del Vallès, Spain) overnight. Tissue was embedded in blocks of paraffin and left to chill in the fridge for a minimum 1 h and stored at room temperature. Subsequently, the excess of paraffin was removed, blocks were precooled 1 h at 4 °C and sections of 4 µm were cut using a microtome Jung Biocut 2035 (Leica Microsystems) and fixed in microscope slides coated with polylysine.

For the hematoxylin-eosin staining, the slides were placed in heating oven for 30 min at 60 °C in order to melt the paraffin and were deparaffinized in xylene twice for 3 min each. After gradual hydration in solutions for 2 min each, with decreasing concentration of ethanol (100%, 96%, 70%) and distilled H_2_O (dH_2_O), the slides were stained with Harris hematoxylin for 3 min 30 s and washed with tap H_2_O for 1 min, changing the water until it appeared to be clean; next, the non-specific stain was removed with hydrochloric acid–alcohol (70% ethanol, 0.35% hydrochloric acid) for 1 s and washed with tap H_2_O for 1 min, 0.03% ammonia H_2_O for 3 s, tap water for 1 min and dH_2_O for 1 min and stained with eosin for 3 min. Next, the slides were dehydrated with increasing concentrations of ethanol (96% 1 min, absolute ethanol for 1 min), ethanol-xylene 1:1 for 5 min, xylene 3 times for 5 min. Finally, the coverslips were mounted on the slides with DPX.

### 2.11. Statistical Analysis

Results are expressed as the mean ± SD or SEM of independent experiments. One-way ANOVA and Tukey’s post hoc analyses, or Kruskal–Wallis with Dunn’s multiple comparison test for in vivo experiments, were carried out with the Statgraphics centurion statistical package. Statistically significant differences, *p* < 0.05, *p* < 0.01, *p* < 0.001, *p* < 0.0001, are represented by *, **, *** and ****, respectively.

## 3. Results

### 3.1. Changes in Cancer Cell Adhesion and Morphology after PG Treatment

The ability of detached melanoma cancer cells treated with PGs (Figure 1A) to adhere again to cell-culture plates was evaluated in order to assess their ability to bind to extracellular-matrix (ECM) components to migrate during the first steps of metastasis or to be able to outgrow during the colonization of a distant site. Therefore, metastatic melanoma SK-MEL-5 cells or mouse melanoma B16F10 cells were treated with different concentrations of PGs for 16 h. Next, they were detached and re-seeded and cellular adhesion was evaluated after 2 h (SK-MEL-5) or 5 h (B16F10). Cellular adhesion sharply decreased after treatment with PGs in both cell lines (Figure 1B). After 24 h treatment with 1 µM of PG or OBX, only around 30% or less of the cells were able to attach to the plate, with PG displaying a more potent effect.

Moreover, changes in cellular morphology were assessed after treatment. In this regard, filopodia are actin-rich membrane protrusions with an important role in modulating cell adhesion and extracellular sensing during cell migration [28]. Therefore, the morphological changes in these structures upon PG treatment were also analyzed (Figure 1C). Melanoma SK-MEL-5 and B16F10 cells were treated with OBX or PG and filopodia were quantified. These structures were reduced, especially after 24 h of treatment with PG and OBX in B16F10 cells (Figure 1C right panel).

**Figure 2:**
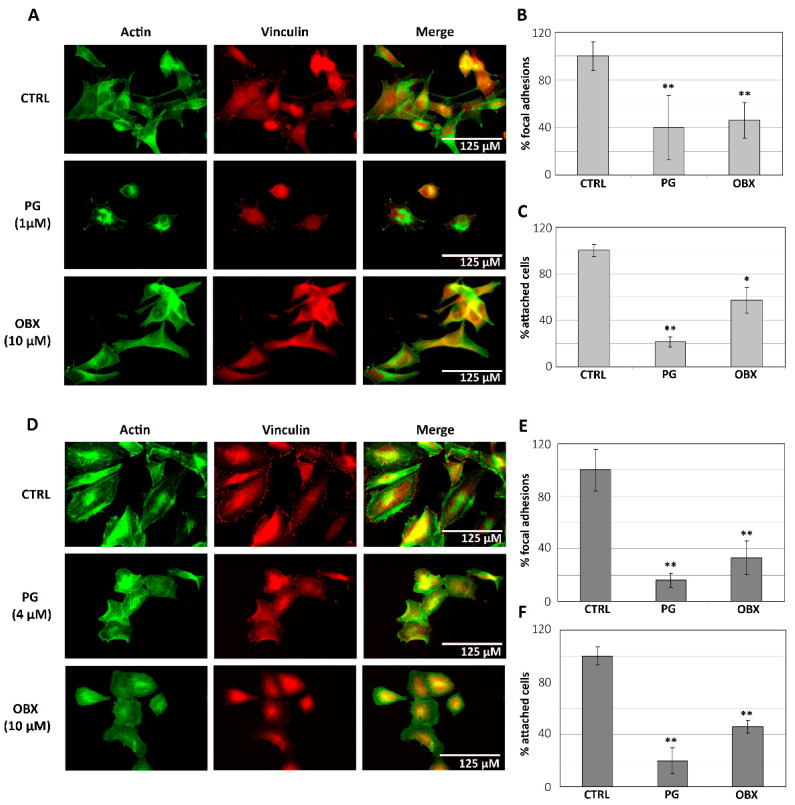
Focal-adhesion evaluation after PG or OBX treatment in SK-MEL-5 (**A**–**C**) and in SK-MEL-28 (**D**–**F**). Immunofluorescence of vinculin (red) and actin (green) shows focal adhesions in SK-MEL-5 (**A**) and SK-MEL-28 (**D**) cells after 3 h of treatment with PG (1 or 4 µM, respectively) or OBX (10 µM). The percentage of focal adhesions (**B**,**E**), as well as the percentage of cell attachment (**C**,**F**), were quantified. Figure shows mean ± SD. Statistical differences from control (CTRL) are shown as ** *p* < 0.01 and * *p* < 0.05.

On the other hand, focal adhesions are large, stable complexes composed of large numbers of proteins, especially paxillin, vinculin and alpha-v beta-3 integrin and function to increase cell adhesion. Therefore, the number of focal adhesions was analyzed by vinculin immunostaining and quantified to complement the previous experiments. Metastatic melanoma SK-MEL-5 and primary tumor melanoma SK-MEL-28 cells were treated with 1 or 4 µM PG, or 10 µM OBX for 3 h. The previous observed decrease in cellular adhesion and filopodia was accompanied by a significant decrease in the number of focal-adhesion structures and a reduction in the number of attached cells in both cell lines (Figure 2).

Altogether, minimal cell adhesion to extracellular matrix, reduced filopodia, as well as a decrease in focal adhesion structures, suggest that treated cancer cells would not be able to efficiently bind to ECM and migrate.

### 3.2. PG Treatment Decreases Cellular Migration

**Figure 3:**
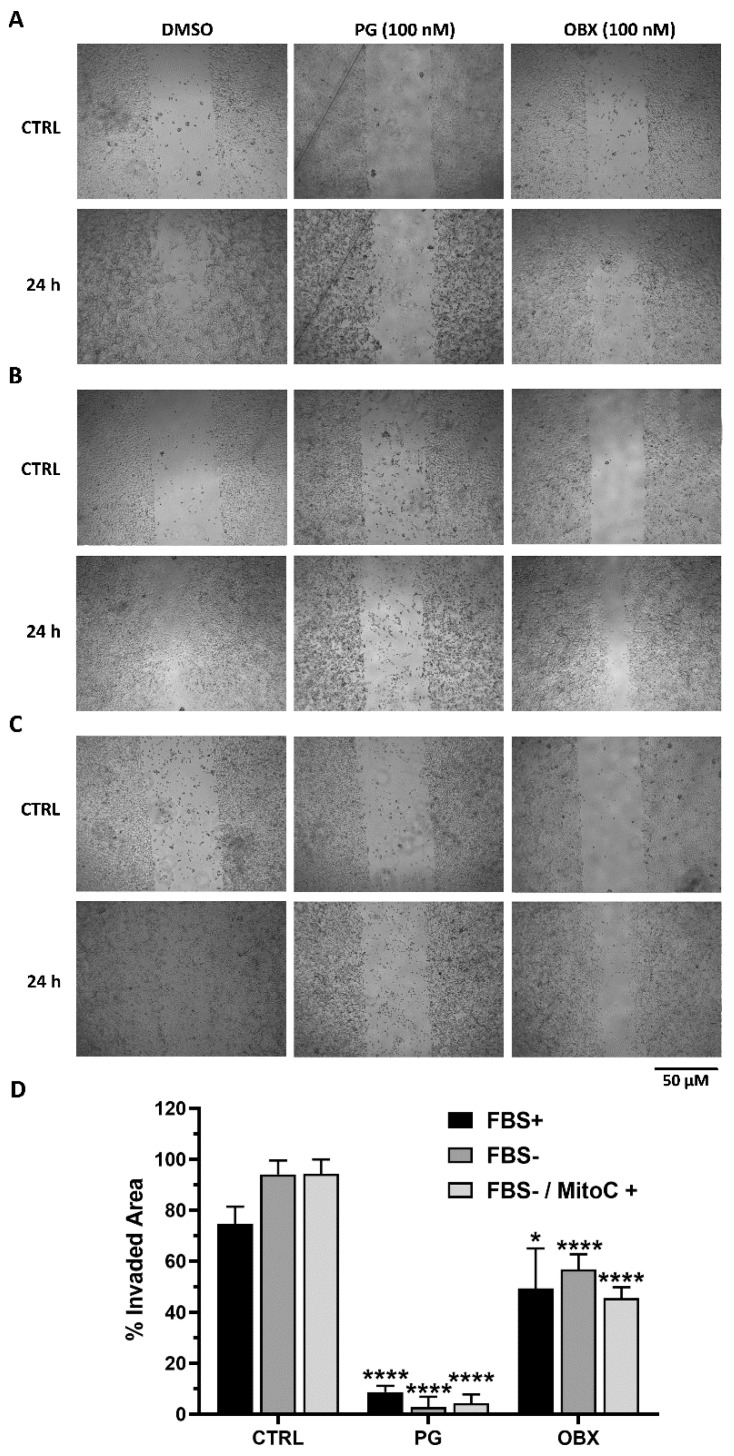
Effects of PG and OBX on B16F10 cell migration. The wound-healing assay was performed in three conditions: complete media with serum (FBS+) (**A**), serum-deprived (FBS-) (**B**) and serum-deprived plus mitomycin (FBS-/MitoC+) treatment (**C**). Cells were treated with 200 nM of PG or OBX for 24 h. Representative images of three independent replicates are shown. (**D**) Quantification of the % invaded area after 24 h of PG and OBX treatment. Figure shows mean ± SD. Statistical differences from control (CTRL) are shown as **** *p* < 0.0001 and * *p* < 0.05.

To deeply characterize the PGs’ antimetastatic effects, especially those related to cell-migration ability, we analyzed PGs’ effects on cancer cells’ motility through the wound-healing assay. The human SK-MEL-5 and -28 melanoma cell lines do not grow to a confluent monolayer; thus, it is not possible to accurately evaluate antimigratory PGs’ effects on these cells through wound-healing assay. Hence, the B16F10 mouse melanoma cells and the other human cancer cell line, HN4, were used to perform this assay. The B16F10 were grown to a confluent monolayer and a scratch was applied with a sterile tip. Next, they were exposed to non-toxic concentrations of PGs in three different experimental conditions: complete media with serum (Figure 3A), serum deprivation (Figure 3B) and serum deprivation plus mitomycin treatment (Figure 3C). After 24 h, the control cells were able to occupy almost all the wounded area, whilst all the PG- and OBX-treated cells experienced a very significant decrease in their ability to migrate under all the experimental conditions (Figure 3). Reduced migration capacity was especially observed after PG treatment in all the conditions (Figure 3D), with cells occupying less than 10% of the wounded area. Similar inhibitory results in cellular migration were obtained in the treated human oral squamous HN4 cells, which also grow forming a monolayer (Supplementary Figure S1).

### 3.3. Metastasis-Related Genes Were Modified by PG Treatment

**Figure 4:**
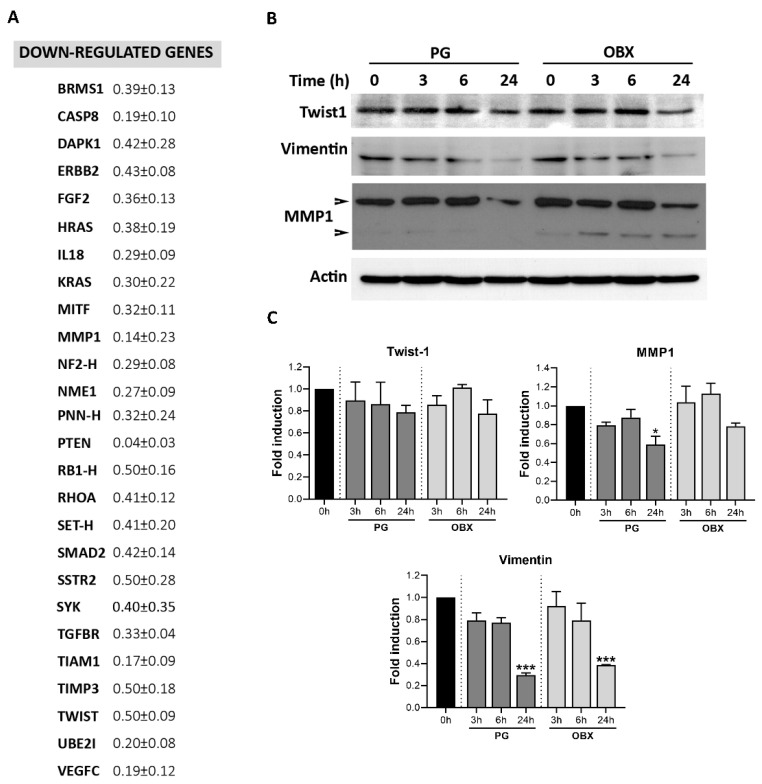
Metastasis-related gene and protein-expression modifications after PG and OBX treatments. (**A**) Gene-expression profiling of SK-MEL-5 melanoma cells after PG treatment (0.5 µM 16 h) was assessed through TLDA technology. (**B**,**C**) Protein-expression levels of metastasis-related proteins twist-1, vimentin and MMP-1 in PG- and OBX (500 nM)-treated SK-MEL-5 cells were assessed through Western blot from 3 to 24 h. Actin expression was used as loading control. Representative images of three independent experiments are shown. Figure shows mean ± SEM. Statistical differences from control 0 h are shown as *** *p* < 0.001 and * *p* < 0.05.

In order to elucidate the molecular mechanisms that were involved in the antimetastatic properties of PGs, we examined the most relevant genes that were involved in this phenomenon. For this purpose, we treated the SK-MEL-5 melanoma cells with PG and analyzed the changes in the expression level of the genes related to metastasis through Taqman Low Density Array (TLDA) technology (see gene-expression profiling results of all genes in Supplementary Table S1). The genes related to invasion (i.e., MMP-1), migration (i.e., IL-18) or epithelial-to-mesenchymal transition (i.e. Twist-1), among others, were significantly down-regulated (Figure 4A). The expression levels of some proteins associated with metastasis, such as twist-1, vimentin and MMP-1, were analyzed and a relevant decrease was observed after 24 h of treatment with 500 nM of PG or OBX (Figure 4B,C).

### 3.4. Obatoclax Shows Antimetastatic Properties In Vivo

**Figure 5:**
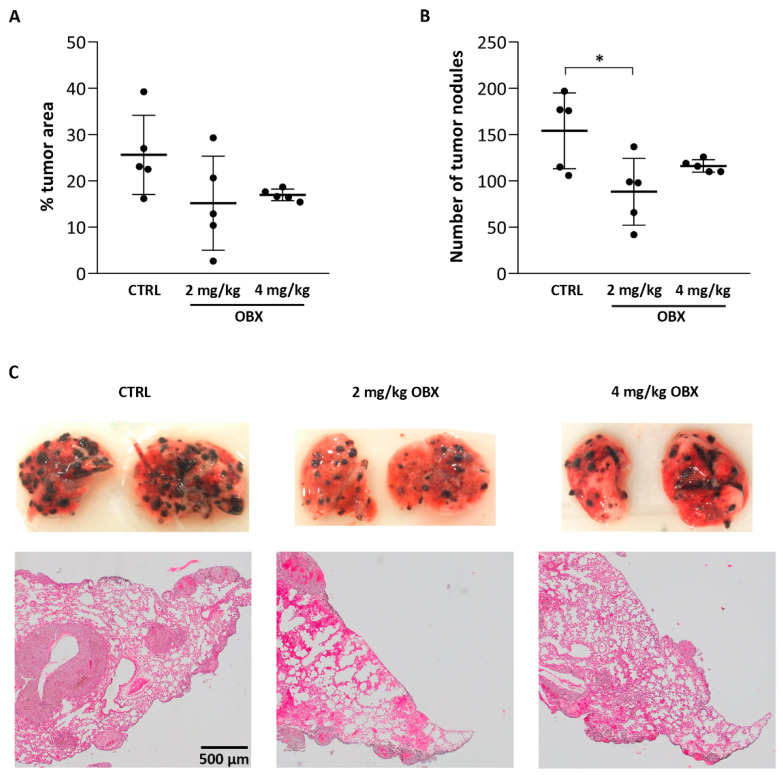
In vivo evaluation of antimetastatic properties of OBX. Mice with lung metastasis were treated daily with different doses of OBX (2 mg/Kg, 4 mg/Kg OBX) for 10 days. (**A**) Quantification of the tumor area in mice treated with OBX or vehicle (control, CTRL). (**B**) Quantification of the number of tumor nodules in control and treated mice. (**C**) Representative images of external photographs of the lungs and hematoxylin-eosin sections from lungs of control and treated mice. Statistical differences from control are shown as * *p* < 0.05.

Finally, we wanted to evaluate OBX’s antimetastatic properties in vivo. For this purpose, B16F10 melanoma cells were inoculated into Balb/c mice through tail-vein injection in order to induce lung metastasis. Different OBX doses (2 mg/Kg and 4 mg/Kg) were administered daily for ten days and the percentage of the tumor area in the lung, as well as the number of external visible lung nodules, were quantified to assess the potential antimetastatic effects of OBX (Figure 5). The quantification of the tumor area (Figure 5A) showed differences between the means of the non-treated and OBX-treated mice, although these were not statistically significant. However, the number of tumor nodules was significantly lower in the mice treated with 2 mg/Kg OBX (Figure 5B). The difference in tumor nodules between control and OBX-treated lungs can be visually appreciated (Figure 5C). Images of lungs from all control and treated mice are shown in Supplementary Figure S2. Finally, the anatomo-pathological analysis of inner tumors, through hematoxylin-eosin staining, corroborated the antimetastatic effects of OBX (Figure 5C).

## 4. Discussion

Metastasis is a multi-step process through which cancer cells can escape from the primary tumor and form new tumors at distant tissues. It is responsible for approximately 90% of cancer-related deaths [1,29], suggesting that targeting metastasis could be a successful therapeutic option. Nevertheless, although targeting metastasis has yielded some results in the prevention of initial metastasis, the preclinical studies or clinical trials performed until now have not considerably improved patient outcomes [30]. Thus, novel anticancer drugs with potent antimetastatic properties are eagerly awaited. Here, we studied the antimetastatic properties of two small molecules pertaining to the anticancer chemical family of Prodiginines: prodigiosin (PG) and, in more depth, the synthetic prodiginine analog Obatoclax (OBX), due to its clinical relevance [10,31].

Tumor cells must be able to adhere to other organs to metastasize [32]. We discovered that the treatment of melanoma cells with PGs sharply reduced cellular adhesion capacity in SK-MEL-5 human metastatic melanoma cells and B16F10 murine melanoma cells. The impairment of adhesion capacity by PGs may hinder melanoma cells from colonizing distant organs, preventing metastasis formation. Similar to our results, a previous study showed a reduced adhesion capacity of highly metastatic human lung cancer cells to ECM after PG treatment [14].

Cell migration, an essential capacity of cells that are going to metastasize, is conditioned by actin-cytoskeleton changes, which leads to the generation of structures such as filopodia and lamellipodia [33]. These changes allow the cancer cell to leave the primary tumor, enter blood or lymphatic vessels and reach the colonization site [34]. In ovarian cancer, for instance, filopodia play a critical role in the migration of cancer cells through the mesothelium (the cell layer that protects peritoneal organs and that should be crossed by cancer cells to invade new organs) [35]. The mechanisms and the important role of these structures in melanoma metastasis has also been described [36,37]. In our study, we found that treatment with both PGs significantly diminished filopodia in melanoma cells, impeding their ability to migrate and, thus, to form metastases. These filopodia-like protrusions appear to be critical not only in driving cancer-cell metastasis, but also in promoting the survival and proliferation of the disseminated carcinoma cells at a secondary organ [38]. Therefore, PG and OBX treatment may significantly compromise the cell viability of disseminated cancer cells, by decreasing the number of filopodia, reducing their metastatic potential. In accordance with our results, nasopharyngeal cancer cells reduced their migration and invasion capacity after PG treatment [39]. Moreover, a previous study found that OBX can reduce cell migration and the adhesion of hepatoblastoma and hepatocellular carcinoma cells to ECM [16]

One of the steps in cell migration [40] is focal-adhesion turnover. Focal adhesions are complexes formed by integrins and growth-factor receptors that facilitate the connection between cells and ECM. This ECM–cell connection is crucial for cells, since it determines cell morphology and cytoplasmic signaling for survival, proliferation, differentiation and motility. Integrins that are in the leading edge of the cancer cell adhere to the ECM, where they initially form short-lived nascent adhesions that develop into new focal adhesion. The assembly and disassembly of focal adhesion and the contractility driven by actin and myosin make possible cell migration [41] and activate multiple signaling cascades [42,43] that promote tumor progression and metastasis. Hence, the significant decrease in focal-adhesion structures induced by PG treatment reinforces the notion that these compounds may limit cancer-cell migration, which is supported by the results obtained by our wound-healing assay.

To deepen our understanding of the antimetastatic properties of PGs, we performed a metastasis-focused gene-expression array that showed significant down-regulation in relevant genes related to epithelial to mesenchymal transition (EMT) (such as twist-1), migration (such as IL-18), or invasion (such as MMP-1). Twist-1 is essential for the metastatic process, since it induces EMT and provides motile and migration properties to the cancer cells. It was previously observed that the inhibition of twist-1 suppresses metastasis [44,45]. On the other hand, patients with colorectal cancer and patients with melanoma who present early metastasis have higher values of IL-18 in their sera than patients without metastasis [46,47]. In endothelial cells, IL-18 upregulates VCAM-1, which enhances metastatic implantation and the spread of cancer cells [48]. Thus, IL-18 inhibition suppresses metastasis because it neutralizes the adhesiveness of cancer cells to the endothelium [49,50]. Finally, matrix metalloproteinases (MMPs) allow the degradation of ECM (specifically collagen), which is one of the primary steps in carcinoma invasion and metastasis [51]. Apart from degrading ECM, it has been observed that MMPs also regulate signaling pathways that control cell growth, inflammation and angiogenesis, which also encourages metastasis [52]. Moreover, MMP-1 activates PAR1, which is associated with invasiveness in melanoma [53,54]. Therefore, all the modifications that we have identified indicate a reduction in the metastatic potential of treated cells.

The antimetastatic properties observed in the different in vitro models were complemented with an in vivo mice model in which melanocytic cells (B16F10 cells) were inoculated by the tail vein to analyze the capacity to establish metastasis in the lungs after OBX treatment. Similarly to previous in vivo PG results [14], the mice treated with OBX showed a significant reduction in the number of nodules formed in the lungs and a decrease in the total tumor area, although the latter parameter did not reach statistical significance. The variability in the results of the control group made it difficult to obtain statistical differences, even though both OBX doses showed a decrease in tumor area and nodule number, which was corroborated by the analyses of the inner-lung tumors through hematoxylin–eosin histological staining. To our knowledge, this is the first time that OBX treatment in vivo has shown antimetastatic effects in a melanoma mice model. Complementing our findings, Lieber et al. showed that hepatoblastoma cells pre-treated in vitro with low doses of OBX prevented cells from metastasizing in the liver when injected in vivo, demonstrating the immune susceptibility produced by OBX treatment [17]. Moreover, previous studies noted that other proapoptotic BH3 mimetic molecules, such as OBX treatment, alone or in combination, exhibit antimetastatic properties in different in vitro and in vivo metastatic-tumor models [55,56,57].

Altogether our findings demonstrate that PG and OBX are able to impair some cellular properties in vitro, such as cell adhesion or migration, which confer metastatic potential to cancer cells. Moreover, some molecular changes were identified in the treated cells, such as down-regulation of key genes related to invasion, migration and epithelial-to-mesenchymal transition, which may be involved in their antimetastatic properties. Finally, our results show significant antimetastatic effects in an in vivo melanoma-induced lung-cancer mouse model after OBX treatment, suggesting that this drug may have promising anticancer effects, not only as a cytotoxic agent, but as an antimetastatic chemotherapeutic agent.

## Data Availability

The data presented in this study are available in this article (and Supplementary Materials).

## Supplementary Materials

The following are available online at https://www.mdpi.com/article/10.3390/pharmaceutics15010097/s1, Figure S1: Effects of PG and OBX on HN4 cell migration. Figure S2: In vivo evaluation of OBX antimetastatic properties. Table S1: Gene-expression profiling of 87 metastasis-related genes after PG treatment.
