# Supplementary material for: Antimetastatic Properties of Prodigiosin and the BH3-Mimetic Obatoclax (GX15-070) in Melanoma
Source: Pharmaceutics. 2022 Dec 28;15(1):97. doi: 10.3390/pharmaceutics15010097 (PMC9862601; doi:10.3390/pharmaceutics15010097)
Supplement: Supplementary file 1 [file pharmaceutics-15-00097-s001.zip › pharmaceutics-2069470-SI.pdf]

Supplementary Materials

Antimetastatic Properties of Prodigiosin and the BH3-Mimetic Obatoclax (GX15-070) in Melanoma

Margarita Espona-Fiedler [1,†], Pilar Manuel-Manresa [1], Cristina Benítez-García [1,2], Pere Fontova [3], Roberto Quesada [3], Vanessa Soto-Cerrato [1,2,*] and Ricardo Pérez-Tomás [1,2,*]

[1] Department of Pathology and Experimental Therapeutics, Faculty of Medicine and Health Sciences, Universitat de Barcelona, 08907 L'Hospitalet de Llobregat, Spain
[2] Molecular Signalling, Oncobell Program, Institut d'Investigació Biomèdica de Bellvitge (IDIBELL), 08908 L'Hospitalet de Llobregat, Spain
[3] Department of Chemistry, Universidad de Burgos, 09001 Burgos, Spain
* Correspondence: vsoto@ub.edu (V.S.-C.); rperez@ub.edu (R.P.-T.)
† Current address: Department of Radiation Sciences, Oncology, Umeå University, SE-901 85 Umeå, Sweden

Supplementary Figures

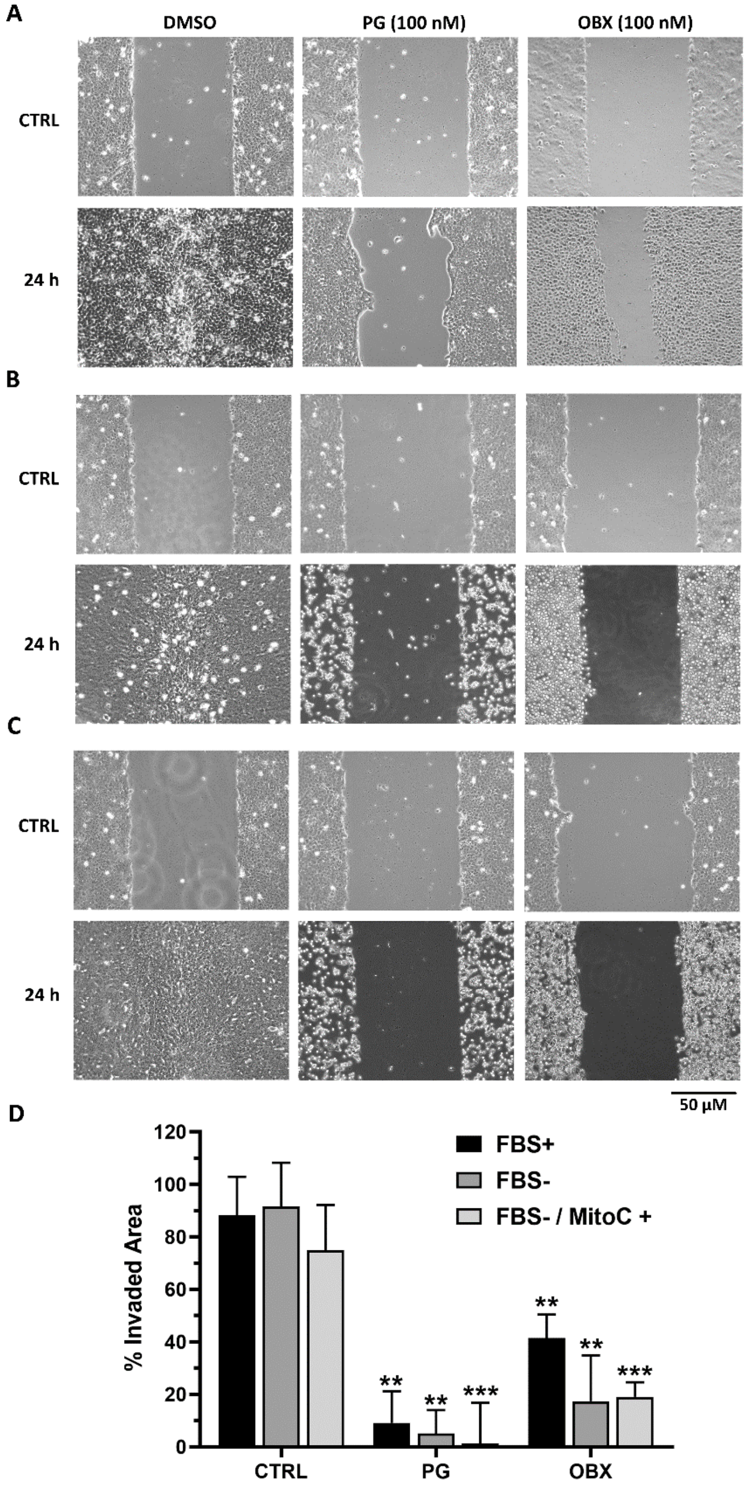

Supplementary Figure S1. Effects of prodigiosin (PG) and obatoclax (OBX) on HN4 cell migration. The wound healing assay was performed in three conditions: complete media with serum (FBS+) (A), serum deprived (FBS-) (B) and serum deprived plus mitomycin (MitoC) treatment (C). Cells were treated with 100 nM of PG or OBX for 24 h. Representative images of three independent replicates are shown. (D) Quantification of the % invaded area after PG and OBX treatment. Figure shows mean ± SD. Statistical differences against control (CTRL) are shown as **** $p < 0.0001$ and * $p < 0.05$.

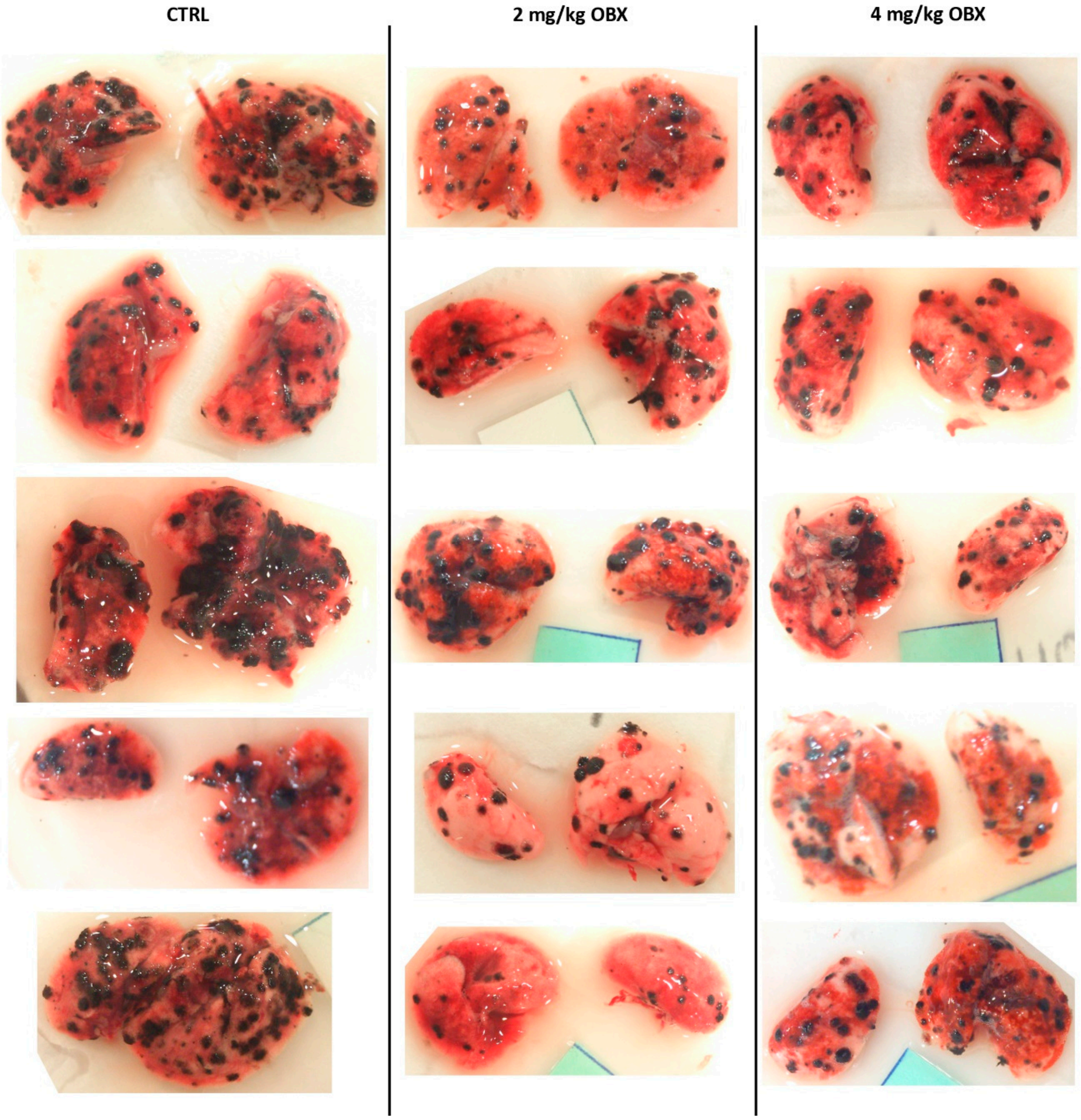

Supplementary Figure S2. In vivo evaluation of obatoclax (OBX) antimetastatic properties. 15 mice were divided into 3 groups. Two groups were treated daily with 2 or 4 mg/kg of OBX for 10 days and the other group was treated with the vehicle (control, CTRL).

Supplementary Tables

Supplementary Table S1. Gene expression profiling of 87 metastasis-related genes after PG treatment. SK-MEL-5 cells were treated with 0.5 μM PG (IC_{25} value) during 16 h and gene expression changes were analyzed by TLDA technology (Applied Biosystems) and DataAssist Software. Mean RQ (Relative Quantitation) of treated cells (IC25 SK5 (RQ)) ± Standard Deviation (SD) are shown in the table below.

DataAssist Software v2.0
Study: SK5 Ct vs IC25
Maximum allowable CT value: 35.0
Include max CT values in calculations: Yes
Exclude outliers among replicates: Yes.
Normalization method: Arithmetic Mean
Selected controls: B2M-Hs99999907_m1
Reference sample: SK5 Ct

		RQ: Relative Quantitation						
**Assay**	**Type**	**CT SK5 (RQ)**	**CT SK5 (RQ Min)**	**CT SK5 (RQ Max)**	**IC25 SK5 (RQ)**	**IC25 SK5 (RQ Min)**	**IC25 SK5 (RQ Max)**	**SD**
**ACTB-Hs99999903_m1**	Candidate Control	**1.00**	0.81	1.23	**0.13**	0.09	0.18	**0.07**
**APC-Hs00181051_m1**	Target	**1.00**	0.81	1.24	**0.70**	0.60	0.81	**0.15**
**B2M-Hs99999907_m1**	Selected Control	**1.00**	0.79	1.26	**1.00**	0.83	1.20	**0.26**
**BRMS1-Hs00363036_m1**	Target	**1.00**	0.79	1.27	**0.39**	0.31	0.49	**0.13**
**CASP8-Hs01018151_m1**	Target	**1.00**	0.85	1.18	**0.19**	0.13	0.27	**0.10**
**CD44-Hs00153304_m1**	Target	**1.00**	0.82	1.22	**1.76**	1.64	1.88	**0.17**
**CD82-Hs00174463_m1**	Target	**1.00**	0.78	1.29	**0.64**	0.48	0.85	**0.26**
**CDH1-Hs00170423_m1**	Target	**1.00**	0.69	1.45	**0.52**	0.36	0.75	**0.28**
**CHI3L1-Hs00609691_m1**	Target	**1.00**	1.00	1.00	**2.55**	2.18	2.99	**0.57**
**CTBP1-Hs00179922_m1**	Target	**1.00**	0.87	1.15	**0.62**	0.46	0.84	**0.27**
**CTNNA1-Hs00426996_m1**	Target	**1.00**	0.83	1.21	**0.55**	0.43	0.71	**0.20**
**CTSK-Hs00166156_m1**	Target	**1.00**	0.72	1.38	**0.56**	0.26	1.20	**0.67**
**CXCL12-Hs00171022_m1**	Target	**1.00**	0.89	1.12	**0.76**	0.59	0.99	**0.28**
**CXCR4-Hs00607978_s1**	Target	**1.00**	0.87	1.15	**4.22**	3.30	5.39	**1.48**
**DAPK1-Hs00234480_m1**	Target	**1.00**	0.95	1.05	**0.42**	0.27	0.67	**0.28**
**DCC-Hs00180437_m1**	Target	**1.00**	0.88	1.13	**2.17**	2.07	2.27	**0.14**
**EPHB2-Hs00362096_m1**	Target	**1.00**	0.87	1.15	**0.59**	0.41	0.85	**0.31**
**ERBB2-Hs00170433_m1**	Target	**1.00**	0.74	1.36	**0.43**	0.38	0.49	**0.08**
**FGF2-Hs00266645_m1**	Target	**1.00**	0.77	1.30	**0.36**	0.29	0.46	**0.13**
**FGFR4-Hs00242558_m1**	Target	**1.00**	0.83	1.21	**1.53**	1.24	1.89	**0.46**
**FN1-Hs00365058_m1**	Target	**1.00**	0.79	1.27	**0.68**	0.53	0.86	**0.23**
**FTH1;MT-CO2;OAF-Hs02596865_g1**	Target	**1.00**	0.79	1.26	**0.75**	0.60	0.93	**0.24**
**FXYD5-Hs00204319_m1**	Target	**1.00**	0.91	1.10	**0.71**	0.53	0.96	**0.30**
**GAPDH-Hs99999905_m1**	Candidate Control	**1.00**	0.84	1.19	**0.23**	0.17	0.31	**0.10**
**GNRH1-Hs00171272_m1**	Target	**1.00**	0.37	2.69	**0.98**	0.61	1.57	**0.68**
**HGF-Hs00300159_m1**	Target	**1.00**	0.69	1.45	**2.43**	1.56	3.78	**1.57**
**HPSE-Hs00180737_m1**	Target	**1.00**	0.75	1.34	**0.94**	0.64	1.40	**0.54**
**HRAS-Hs00610483_m1**	Target	**1.00**	0.87	1.15	**0.38**	0.27	0.54	**0.19**
**HTATIP2-Hs00185131_m1**	Target	**1.00**	0.85	1.18	**0.82**	0.63	1.07	**0.31**
**IGF1-Hs00153126_m1**	Target	**1.00**	0.87	1.15	**0.86**	0.63	1.19	**0.39**
**IL18-Hs00155517_m1**	Target	**1.00**	0.70	1.44	**0.29**	0.24	0.36	**0.09**
**IL1B-Hs00174097_m1**	Target	**1.00**	0.59	1.70	**1.31**	1.06	1.62	**0.40**
**ITGA7-Hs00174397_m1**	Target	**1.00**	0.58	1.72	**1.54**	1.27	1.87	**0.43**
**ITGB3-Hs00173978_m1**	Target	**1.00**	0.83	1.21	**1.12**	0.91	1.38	**0.33**
**KRAS-Hs00270666_m1**	Target	**1.00**	0.72	1.39	**0.30**	0.18	0.49	**0.22**
**LAMB1-Hs00158620_m1**	Target	**1.00**	0.86	1.17	**0.63**	0.57	0.69	**0.08**

**MCAM-Hs00174838_m1**	Target	**1.00**	0.79	1.26	**0.70**	0.54	0.91	**0.26**
**MET-Hs00179845_m1**	Target	**1.00**	0.85	1.17	**2.14**	1.81	2.54	**0.52**
**MGAT5-Hs00159136_m1**	Target	**1.00**	0.82	1.21	**0.59**	0.51	0.68	**0.12**
**MITF-Hs00165156_m1**	Target	**1.00**	0.76	1.32	**0.32**	0.25	0.40	**0.11**
**MMP1-Hs00233958_m1**	Target	**1.00**	0.65	1.54	**0.14**	0.05	0.38	**0.23**
**MMP10-Hs00233987_m1**	Target	**1.00**	1.00	1.00	**2.47**	2.24	2.72	**0.34**
**MMP14-Hs01037009_g1**	Target	**1.00**	0.78	1.28	**0.86**	0.68	1.08	**0.28**
**MMP2-Hs00234422_m1**	Target	**1.00**	0.61	1.63	**0.55**	0.28	1.09	**0.57**
**MTA1-Hs00183042_m1**	Target	**1.00**	0.86	1.17	**0.98**	0.75	1.26	**0.36**
**MTSS1-Hs00207341_m1**	Target	**1.00**	0.78	1.28	**0.65**	0.56	0.76	**0.14**
**MYC-Hs00153408_m1**	Target	**1.00**	0.83	1.20	**0.52**	0.39	0.69	**0.21**
**NCAM1-Hs00169851_m1**	Target	**1.00**	0.65	1.54	**0.84**	0.82	0.85	**0.02**
**NF2-Hs00738978_m1**	Target	**1.00**	0.82	1.22	**0.29**	0.23	0.35	**0.08**
**NME1-Hs02621161_s1**	Target	**1.00**	0.76	1.32	**0.27**	0.21	0.34	**0.09**
**NR4A3-Hs00235001_m1**	Target	**1.00**	0.82	1.21	**0.99**	0.84	1.16	**0.22**
**PECAM1-Hs00169777_m1**	Target	**1.00**	0.84	1.19	**2.45**	1.90	3.16	**0.89**
**PNN-Hs00170192_m1**	Target	**1.00**	0.76	1.31	**0.32**	0.20	0.54	**0.24**
**PTEN-Hs01920652_s1**	Target	**1.00**	0.72	1.38	**0.04**	0.02	0.06	**0.03**
**RB1-Hs00153108_m1**	Target	**1.00**	0.69	1.44	**0.50**	0.41	0.63	**0.16**
**RET-Hs00240887_m1**	Target	**1.00**	0.66	1.52	**1.10**	0.77	1.57	**0.57**
**RHOA-Hs00357608_m1**	Target	**1.00**	0.78	1.27	**0.41**	0.33	0.50	**0.12**
**RHOC-Hs00733980_m1**	Target	**1.00**	0.79	1.27	**0.84**	0.49	1.46	**0.69**
**RORB-Hs00199445_m1**	Target	**1.00**	0.51	1.95	**1.15**	1.01	1.31	**0.21**
**RYBP-Hs00171928_m1**	Target	**1.00**	0.79	1.26	**0.96**	0.81	1.14	**0.24**
**SERPINE1-Hs00167155_m1**	Target	**1.00**	0.77	1.29	**1.47**	0.77	2.81	**1.45**
**SET-Hs00853870_g1**	Target	**1.00**	0.84	1.20	**0.41**	0.29	0.57	**0.20**
**SMAD2-Hs00183425_m1**	Target	**1.00**	0.81	1.23	**0.42**	0.33	0.52	**0.14**
**SMAD4-Hs00232068_m1**	Target	**1.00**	0.84	1.19	**0.66**	0.58	0.76	**0.13**
**SNCG-Hs00268306_m1**	Target	**1.00**	0.45	2.20	**1.64**	1.13	2.39	**0.89**
**SSTR2-Hs00265624_s1**	Target	**1.00**	0.42	2.39	**0.50**	0.34	0.73	**0.28**
**SYK-Hs00374292_m1**	Target	**1.00**	0.69	1.44	**0.40**	0.22	0.72	**0.35**
**TCF20-Hs00390028_m1**	Target	**1.00**	0.77	1.30	**0.51**	0.41	0.64	**0.16**
**TFRC-Hs99999911_m1**	Candidate Control	**1.00**	0.88	1.14	**0.99**	0.80	1.23	**0.31**
**TGFB1-Hs99999918_m1**	Target	**1.00**	0.76	1.31	**0.57**	0.46	0.70	**0.17**
**TGFBR2-Hs00559661_m1**	Target	**1.00**	0.82	1.23	**0.33**	0.30	0.36	**0.04**
**TIAM1-Hs00180075_m1**	Target	**1.00**	0.86	1.17	**0.17**	0.12	0.25	**0.09**
**TIMP1-Hs00171558_m1**	Target	**1.00**	0.85	1.17	**0.66**	0.50	0.88	**0.27**
**TIMP2-Hs00234278_m1**	Target	**1.00**	0.82	1.22	**1.06**	0.78	1.44	**0.47**
**TIMP3-Hs00165949_m1**	Target	**1.00**	0.81	1.24	**0.50**	0.39	0.65	**0.18**
**TWIST1-Hs00361186_m1**	Target	**1.00**	0.77	1.30	**0.50**	0.44	0.58	**0.09**
**UBC-Hs00824723_m1**	Candidate Control	**1.00**	0.83	1.21	**0.94**	0.73	1.21	**0.34**
**UBE2I-Hs00163336_m1**	Target	**1.00**	0.78	1.28	**0.20**	0.16	0.27	**0.08**
**VEGF-Hs00900054_m1**	Target	**1.00**	0.81	1.24	**2.31**	1.90	2.81	**0.64**
**VEGFC-Hs00153458_m1**	Target	**1.00**	0.86	1.16	**0.19**	0.12	0.29	**0.12**
